# Quality of life associated with breathlessness in the multinational Burden of Obstructive Lung Disease (BOLD) study: A cross-sectional analysis

**DOI:** 10.1080/25310429.2025.2470566

**Published:** 2025-04-02

**Authors:** Alexander Müller, Emiel F. M. Wouters, Peter Burney, James Potts, Joao Cardoso, Mohammed Al Ghobain, Michael Studnicka, Daniel Obaseki, Asma Elsony, Kevin Mortimer, David Mannino, Rain Jõgi, Rana Ahmed, Asaad Nafees, Maria Fatima Rodrigues, Cristina Bárbara, Rune Nielsen, Thorarinn Gíslason, Hamid Hacene Cherkaski, Karima El Rhazi, Christer Janson, Mahesh Padukudru Anand, Sanjay Juvekar, Herminia Brites Dias, Frits M. E. Franssen, Dhiraj Agarwal, Sylvia Hartl, Terence Seemungal, Stefanni Nonna Paraguas, Imed Harrabi, Meriam Denguezli, Abdul Rashid, Gregory Erhabor, Mohammed El Biaze, Parvaiz Koul, Daisy J. A. Janssen, André F. S. Amaral, on behalf of the BOLD Collaborative Research Group

**Affiliations:** a Ludwig Boltzmann Institute for Lung Health, Vienna, Austria; b Department of Health Services Research, Care and Public Health Research Institute, Faculty of Health Medicine and Life Sciences, Maastricht University, Maastricht, The Netherlands; c Faculty of Medicine, Sigmund Freud Private University, Vienna, Austria; d Department of Respiratory Medicine, Maastricht University Medical Center, Maastricht, The Netherlands; e National Heart and Lung Institute, Imperial College London, London, UK; f Pulmonology Department, Centro Hospitalar Universitário de Lisboa Central, Lisboa, Portugal; g NOVA Medical School, Nova University Lisbon, Lisboa, Portugal; h King Abdullah International Medical Research Center, King Saud ben Abdulaziz University for Health Science, Riyadh, Saudi Arabia; i Department of Respiratory Medicine, University Hospital, Paracelsus Medical University, Salzburg, Austria; j Department of Medicine, Obafemi Awolowo University, Ile-Ife, Nigeria; k Faculty of Medicine, University of British Columbia, Vancouver, Canada; l The Epidemiological Laboratory, Khartoum, Sudan; m University of Cambridge, Cambridge, UK; n Liverpool University Hospitals NHS Foundation Trust, Liverpool, UK; o University of Kentucky, Lexington, KY, USA; p COPD Foundation, Miami, FL, USA; q Lung Clinic, Tartu University Hospital, Tartu, Estonia; r Aga Khan University, Karachi, Pakistan; s Pulmonology Department, Santa Maria Local Health Unit, Lisbon, Portugal; t Institute of Environmental Health, Lisbon Medical School, Lisbon University, Lisbon, Portugal; u Instituto de Saúde Ambiental, Faculdade de Medicina, Universidade de Lisboa, Lisbon, Portugal; v Serviço de Pneumologia, Unidade Local de Saúde de Santa Maria, Lisbon, Portugal; w Department of Clinical Science, University of Bergen, Bergen, Norway; x Department of Thoracic Medicine, Haukeland University Hospital, Bergen, Norway; y Faculty of Medicine, University of Iceland, Reykjavik, Iceland; z Department of Sleep, Landspitali – The National University Hospital of Iceland, Reykjavik, Iceland; aa Department of Pneumology, Faculty of Medicine, University Badji Mokhtar of Annaba, Annaba, Algeria; bb Department of Epidemiology and Public Health, Epidemiology and Research in Health Sciences Laboratory, Faculty of Medicine, Pharmacy and Dentistry, Sidi Mohamed Ben Abdillah University, Hassan II University Hospital Center of Fes, Fes, Morocco; cc Department of Medical Sciences: Respiratory, Allergy and Sleep Research, Uppsala University, Uppsala, Sweden; dd Department of Respiratory Medicine, JSS Medical College, JSSAHER, Mysore, India; ee Vadu Rural Health Program, KEM Hospital Research Centre, Pune, India; ff Dr. D.Y. Patil Medical College, Hospital and Research Centre, Dr. D.Y. Patil University, Pimpri, Pune, India; gg Lisbon School of Health Technology, Polytechnic of Lisbon, Lisbon, Portugal; hh Department of Research and Development, Ciro, Horn, The Netherlands; ii Faculty of Medical Sciences, University of West Indies, St Augustine, Trinidad and Tobago; jj Philippine College of Chest Physicians, Manila, Philippines; kk Ibn El Jazzar Faculty of Medicine of Sousse, University of Sousse, Sousse, Tunisia; ll Faculté de Médecine de Sousse, Université de Sousse, Sousse, Tunisia; mm Faculté de Médecine Dentaire de Monastir, Université de Monastir, Monastir, Tunisia; nn Royal College of Surgeons in Ireland and University College Dublin Malaysia Campus, Penang, Malaysia; oo Department of Respiratory Medicine, Faculty of Medicine, Mohammed Ben Abdellah University, Fes, Morocco; pp Department of Pulmonary Medicine, Sher-i-Kashmir Institute of Medical Sciences, Srinagar, India; qq Department of Family Medicine, Care and Public Health Research Institute, Faculty of Health Medicine and Life Sciences, Maastricht University, Maastricht, The Netherlands; rr NIHR Imperial Biomedical Research Centre, London, UK

**Keywords:** Dyspnoea, breathlessness, quality of life

## Abstract

**Introduction:**

Evidence of an association between breathlessness and quality of life from population-based studies is limited. We aimed to investigate the association of both physical and mental quality of life with breathlessness across several low-, middle- and high-income countries.

**Methods:**

We analysed data from 19 714 adults (31 sites, 25 countries) from the Burden of Obstructive Lung Disease (BOLD) study. We measured both mental and physical quality of life components using the SF-12 questionnaire, and defined breathlessness as grade ≥2 on the modified Medical Research Council scale. We used multivariable linear regression to assess the association of each quality-of-life component with breathlessness. We pooled site-specific estimates using random-effects meta-analysis.

**Results:**

Both physical and mental component scores were lower in participants with breathlessness compared to those without. This association was stronger for the physical component (coefficient = −7.59; 95%CI −8.60, −6.58; I^2^ = 78.5%) than for the mental component (coefficient = −3.50; 95%CI −4.36, −2.63; I^2^ = 71.4%). The association between physical component and breathlessness was stronger in high-income countries (coefficient = −8.82; 95%CI −10.15, −7.50). Heterogeneity across sites was partly explained by sex and tobacco smoking.

**Conclusion:**

Quality of life is worse in people with breathlessness, but this association varies widely across the world.

## Introduction

Breathlessness, also referred to as dyspnoea, is defined as an individual experience of breathing discomfort caused by different pathophysiological mechanisms.^1^ The risk factors for breathlessness in adults include reduced lung function, high body mass index (BMI), older age, female sex, history of smoking and reduced physical activity.^2–6^ Additionally, breathlessness is associated with various conditions such as respiratory disease, cardiovascular disease (CVD), malignancy, and neuromuscular disease.^7^ Prior research conducted by Currow and colleagues has shown that breathlessness has a significant impact on quality of life in a population-based sample of Australian adults. A strong association was reported between breathlessness and both physical and mental quality of life component scores.^8^ The impact on daily activities seems to be greatest in people living with chronic breathlessness for two to six years.^9^ Currently, data on the association between brethlessness and quality of life are limited to studies from regions with a high gross national income.^8,10–12^ Janson and colleagues examined the association of quality of life and COPD in the Burden of Obstructive Lung Disease (BOLD) study. They found that breathlessness is an important determinant of quality of life.^11^ However, it should be noted that most of the data presented in their study were drawn from high-income regions. It has previously been reported that a higher socioeconomic status is related to better quality of life in patients with chronic lung disease.^13^ Therefore, the lack of data from low- and middle-income countries represents an important limitation of previous research on the impact of breathlessness on quality of life. This study aimed to address this gap by: (1) providing additional data on breathlessness and quality of life including sites from several world regions; (2) quantifying the association between breathlessness and both physical and mental quality of life components; and (3) identifying potential differences in the strength of this association among participants from high-income countries compared to participants from low- and middle-income countries.

## Methods

### Study design

Data from the BOLD study were analysed in this study. The design and rationale of the BOLD study have been previously published.^14^ In summary, non-institutionalised adults aged 40 years and above were recruited from 41 sites, across 34 countries. They provided self-reported information on pre-existing respiratory or cardiovascular diagnoses, respiratory symptoms, health status, and exposure to potential risk factors, including tobacco smoking. Lung function was assessed using spirometry (EasyOne, ndd Medizintechnik AG). Measurements post bronchodilation were taken after the inhalation of 200 µg of albuterol/salbutamol. The quality of these measurements was assessed based on the American Thoracic Society acceptability and reproducibility criteria.^15^ All measurements and questionnaires were conducted in the local language and administered by trained staff. Ethics approval was obtained for all study sites from the local ethics committees, and all participants provided informed consent.

### Dyspnoea

The 5-item modified Medical Research Council (mMRC) scale was used for the assessment of breathlessness. The mMRC items are described as follows: Grade 0 – breathlessness only with strenuous exercise; Grade 1 – breathlessness when hurrying on level ground or up a slight hill; Grade 2 – breathlessness when walking at own pace on the level; Grade 3 – breathlessness when walking 100 yards or for a few minutes; Grade 4 – too short of breath when leaving the house or short of breath when dressing or undressing. For this study, clinically relevant breathlessness was defined as a mMRC grade 2 or higher in accordance with previously published recommendations.^16^

### Quality of life

Quality of life was evaluated using the short form 12 (SF-12) quality of life questionnaire in 31 of the 41 sites included in the BOLD study. These 31 sites were included in the present analysis. The SF-12 questionnaire comprises 12 items which collectively assess eight domains of quality of life (physical functioning; role-physical; bodily pain; general health, vitality; social functioning; role-emotional; mental health). The questionnaire was translated into the local languages according to the International Quality of Life Assessment Project method.^17^ The SF-12 questionnaire results are divided into a physical and a mental component score. A score of 50 (SD 10) indicates the normal value based on reference populations and higher values indicate higher quality of life.^18^

### Statistical analyses

Prevalence estimates for breathlessness were calculated and SF-12 component scores were summarised descriptively. To estimate the association between quality-of-life scores and breathlessness, we used multivariable linear regression models for each study site. The potential confounders included in the regression model were age, sex, CVDs, diabetes, hypertension, smoking status, BMI and FVC and FEV_1_/FVC below the lower limit of normal (LLN), based on the reference equations for Caucasians from the US National Health and Nutrition Examination Survey (NHANES) III.^19^ Prevalence estimates and regression coefficients were corrected for sampling weights to account for different sampling strategies and response rates. Estimates from each study site were pooled and summarised based on gross national income (high-income, low- and middle-income) according to World Bank Group data^20^ using random-effects meta-analysis. Results were also stratified by sex and smoking status. *I*^*2*^ statistic was used to explore heterogeneity across sites. All statistical analyses were conducted using Stata version 17 (StataCorp, USA), and results were considered significant at a p-value of less than 0.05.

## Results

**Figure 1.**
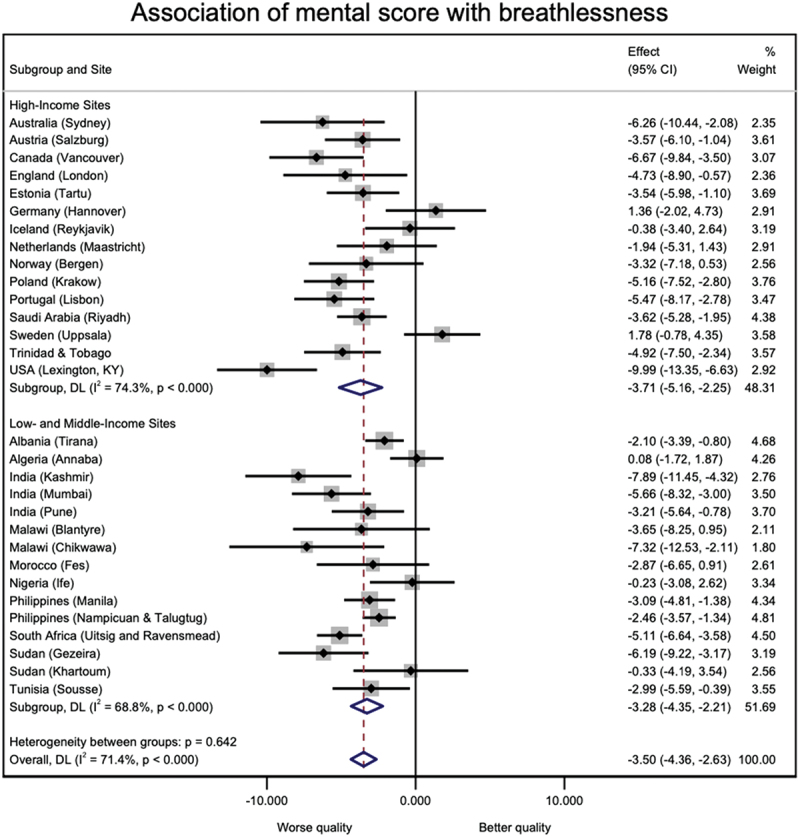
Association of SF-12 mental component score with breathlessness in high income and low/middle income sites.

**Figure 2.**
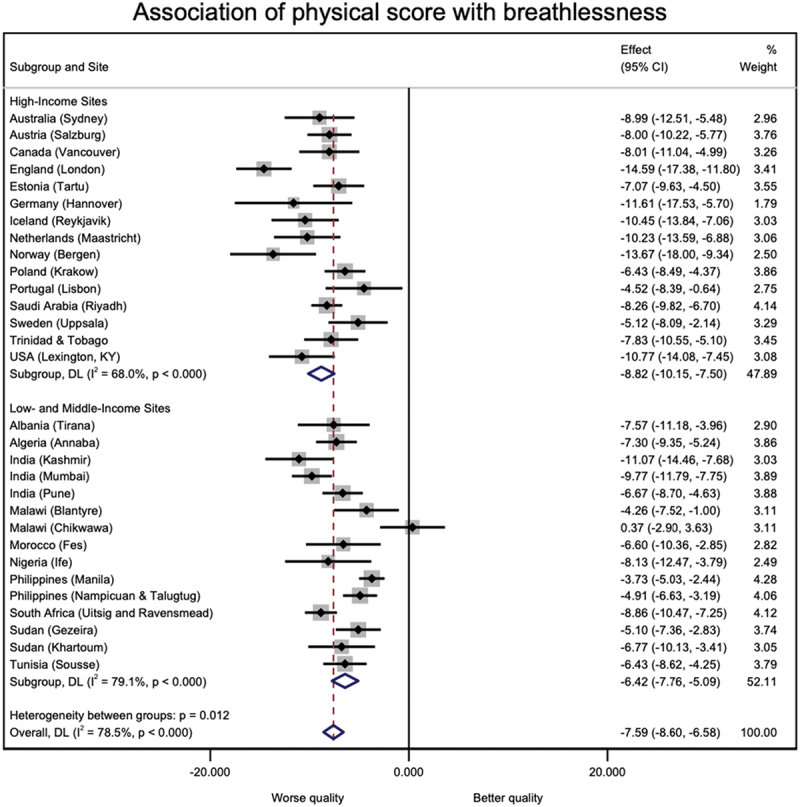
Association of SF-12 physical component score with breathlessness in high income and low/middle income sites.

19,714 participants from 31 sites from the BOLD baseline survey were included in this study. The sites were distributed over 25 countries and five continents. Table 1 shows the distribution of several characteristics of the participants across study sites. The mean age of the study participants was 54.6 years (SD 11.0), 49.6% were males, and most have never smoked. Table 2 shows the prevalence estimates for breathlessness (mMRC ≥2) and the mean component scores of the SF-12 questionnaire for each study site and separated by participants with and without dyspnoea. The prevalence of dyspnoea ranged from 0.0% in Mysore (India) to 28.8% in Nampicuan-Talugtug (Philippines), with a mean prevalence estimate across sites of 11.1% (SD 7.8). Mental component scores ranged from 41.8 (SD 8.4) in Fes (Morocco) to 58.4 (SD 7.1) in Mysore (India). Physical component scores ranged from 43.9 (SD 10.6) in Krakow (Poland) to 53.9 (SD 3.9) in Blantyre (Malawi). The estimates for the associations between mental and physical quality of life component scores are shown in Figures 1 and 2, respectively. Both mental (beta = −3.50, 95%CI −4.36 to −2.63, I^2^ = 71.4%) and physical (beta = −7.59, 95%CI −8.60 to −6.58, I^2^ = 78.5%) quality of life component scores were lower in participants with breathlessness. This association was stronger for the physical component, particularly in high-income countries. Heterogeneity in these associations was high across study sites. For further investigation of this heterogeneity, the meta-analyses were stratified by sex and smoking status. The results of these stratified analyses can be found in figures S1 to S4 of the supplementary material. Sex (females: I^2^ = 90.3% for mental component, I^2^ = 63.5% for physical component; males: I^2^ = 46.3% for mental component, I^2^ = 86.7% for physical component) and smoking status (ever smokers: I^2^ = 57.0% for mental component, I^2^ = 72.6% for physical component; ever smokers: I^2^ = 85.9% for mental component, I^2^ = 70.0% for physical component) can partly explain heterogeneity in the association between quality of life component scores and breathlessness across study sites.

**Table 1.** Characteristics of participants across the BOLD study sites.

	n	Males, %	Age, mean (sd)	BMI, mean (sd)	Never Smokers, %	Cardiovascular disease, %	Diabetes, %	Hypertension, %	Restriction*, %	Obstruction**, %
Albania (Tirana)	887	49.5	55.1 (11.8)	27.5 (4.1)	63.4	3.4	6.9	22.1	15.1	7.4
Algeria (Annaba)	828	51	53.4 (11.1)	28.1 (5.6)	60.3	6.4	14.2	22.2	27.1	6.2
Australia (Sydney)	494	46.8	57.9 (12.5)	27.7 (5)	47.6	12.7	7.8	31.1	12.2	9.7
Austria (Salzburg)	1202	46.8	58.8 (12)	26.2 (4.1)	46.7	13.6	5.9	29.9	9.2	16.9
Canada (Vancouver)	793	47.9	56.3 (12.4)	26.7 (5)	42.7	13.4	7.4	20.2	8.3	12.7
England (London)	647	45.6	57.5 (12.3)	27.1 (4.9)	34.8	7.7	6.5	33.8	16.5	17
Estonia (Tartu)	567	40.5	58.6 (11.9)	28.3 (5.3)	54.5	33.6	6.2	36.7	8.5	5.9
Germany (Hannover)	529	47	56.7 (10.9)	26.6 (4.3)	39.6	13.5	4.8	33.2	7.1	8.2
Iceland (Reykjavik)	726	52	56.6 (11.7)	27.8 (4.9)	33.7	15.3	4.2	31.7	12.5	11.1
India (Kashmir)	757	51.6	51.4 (10.5)	22.6 (3.7)	47.6	1.2	2.6	26.9	28.1	16.2
India (Mumbai)	434	62.9	52.6 (9.5)	23.9 (4.1)	90	2.4	5.2	10.6	67.7	6.5
India (Mysore)	592	42.3	46.5 (6.9)	24.7 (3.8)	90.4	0.2	16.9	17	79	7.9
India (Pune)	831	59.9	52.2 (10)	22.1 (3.9)	87.5	1.4	2.2	5.2	66.1	5.8
Malawi (Blantyre)	359	54.4	50.2 (9.1)	24.4 (5)	83.8	2.8	5.9	16.1	45.8	6.4
Malawi (Chikwawa)	381	56.9	51.3 (9.5)	21.8 (3.8)	70.8	1.2	1.3	1.6	36.9	12.2
Morocco (Fes)	475	60.4	52.9 (11.6)	27.2 (4.8)	64.6	2.8	9.1	21.9	18.5	6.8
Netherlands (Maastricht)	576	47.6	58.2 (11.8)	27.4 (4.5)	35.8	17.8	7.1	29.8	9.8	18.3
Nigeria (Ife)	841	55.1	52.5 (10.9)	25 (5.1)	85.4	0.2	0.6	1.4	71.9	7
Norway (Bergen)	575	49.1	56.5 (12)	26.4 (4.2)	35.4	10.8	4	25.3	8.8	11
Philippines (Manila)	825	46.5	52.4 (10.9)	24.5 (4.7)	43.2	9.3	6.1	23.6	63.7	8.6
Philippines (Nampicuan-Talugtug)	722	49.4	54.2 (10.6)	21.6 (4.1)	44.6	8.7	2.5	20.7	56.7	15.2
Poland (Krakow)	480	49.8	55.1 (11.2)	27.6 (4.7)	38.5	30.4	10.2	40.4	9	13.8
Portugal (Lisbon)	564	48	57.1 (11.7)	27.7 (4.5)	55	10.8	8.9	29.1	10.2	8
Saudi Arabia (Riyadh)	609	54.9	49.5 (7.5)	30.9 (5.7)	73	5.2	27.4	24	51.3	2.8
South Africa (Uitsig-Ravensmead)	766	44.2	52.9 (10.1)	27.5 (7.3)	30.4	11.4	11.4	34.8	46	18.9
Sudan (Gezeira)	386	56.6	52.1 (10.2)	26.9 (17.3)	72.8	0.3	6.4	9.4	58.4	5.9
Sudan (Khartoum)	479	53.1	53.1 (10.9)	26.3 (6.3)	78.3	1.9	8.7	20.6	68	10.1
Sweden (Uppsala)	489	47.6	58.3 (11.2)	26.8 (4.3)	40.9	10.3	3	26.6	8.7	7.8
Trinidad and Tobago	1051	50	54.7 (10.7)	28.7 (9.3)	70.2	5.8	14.8	27.9	73.7	5.9
Tunisia (Sousse)	433	60.7	50.5 (9.1)	27.8 (5.2)	47.2	4	7	14.1	23.2	5.2
USA (Lexington, KY)	416	46.4	56.1 (11.5)	30.4 (6.3)	39.1	24.2	15.7	43.9	23.2	12.4

*Restriction defined as FVC<LLN; **Obstruction defined as FEV1/FVC<LLN.

**Table 2.** Prevalence of breathlessness and mean SF-12 scores for mental and physical quality of life across BOLD study sites.

			Mental component score, mean (sd)			Physical component score, mean (sd)		
Study site	n	Breathlessness (%)	All participants	With breathlessness	Without breathlessness	All participants	With breathlessness	Without breathlessness
Albania (Tirana)	887	9.2	51.4 (5.9)	45.6 (6.6)	52.0 (5.5)	51.5 (6.8)	39.0 (6.5)	52.7 (5.4)
Algeria (Annaba)	828	12.3	49.3 (7.2)	49.3 (7.7)	49.3 (7.1)	49.7 (8.8)	39.5 (9.4)	51.2 (7.7)
Australia (Sydney)	494	7.3	51.6 (9.6)	46.4 (12.6)	52.1 (9.2)	51.0 (8.7)	39.2 (10.1)	52.0 (7.9)
Austria (Salzburg)	1202	8	54.2 (8.6)	50.6 (11.1)	54.5 (8.3)	50.4 (7.5)	40.5 (9.7)	51.3 (6.6)
Canada (Vancouver)	793	6.7	50.8 (9.6)	44.9 (11.5)	51.2 (9.4)	51.6 (9.0)	40.0 (10.8)	52.5 (8.2)
England (London)	647	14.4	48.8 (11)	45.2 (12.3)	49.4 (10.7)	48.9 (10.3)	34.2 (9.9)	51.4 (8.1)
Estonia (Tartu)	567	13.7	52.0 (8.7)	48.2 (9.8)	52.6 (8.4)	47.2 (9.2)	38.2 (9.6)	48.7 (8.3)
Germany (Hannover)	529	3.8	55.9 (7.5)	59.0 (7.8)	55.8 (7.5)	48.6 (7.9)	33.9 (13.1)	49.1 (7.0)
Iceland (Reykjavik)	726	8.6	53.6 (8.9)	52.9 (11.2)	53.7 (8.6)	50.5 (9.3)	38.2 (12.4)	51.6 (8.1)
India (Kashmir)	757	4.9	51.7 (6.3)	41.8 (6.6)	52.2 (5.9)	51.2 (6.6)	36.8 (8.8)	52.0 (5.6)
India (Mumbai)	434	9.8	58.0 (6.6)	50.5 (7.8)	58.9 (5.9)	52.3 (7.1)	41.4 (4.8)	53.5 (6.2)
India (Mysore)	592	0	58.4 (7.1)	-	58.4 (7.1)	53.3 (4.1)	-	53.3 (4.1)
India (Pune)	831	6.6	49.3 (7.4)	45.9 (8.6)	49.5 (7.3)	50.1 (6.6)	41.9 (7.2)	50.7 (6.2)
Malawi (Blantyre)	359	1.9	54.0 (8.2)	48.5 (6.9)	54.1 (8.2)	53.9 (3.9)	49.8 (4.3)	54.0 (3.9)
Malawi (Chikwawa)	381	1.4	56.0 (7.9)	48.2 (9.4)	56.1 (7.8)	52.3 (4.1)	52.1 (5.2)	52.3 (4.1)
Morocco (Fes)	475	14.8	41.8 (8.4)	38.5 (7.3)	42.3 (8.5)	50.8 (9.2)	43.3 (12.1)	52.1 (7.9)
Netherlands (Maastricht)	576	10.1	53.5 (9.4)	51.2 (11.9)	53.8 (9.1)	50.1 (9.0)	38.3 (11.3)	51.4 (7.7)
Nigeria (Ife)	841	3.2	55.2 (9.7)	53.8 (8.9)	55.3 (9.8)	45.5 (8.3)	36.8 (10.4)	45.8 (8.0)
Norway (Bergen)	575	5.3	54.2 (8.9)	51.8 (10.9)	54.4 (8.8)	50.8 (8.7)	34.7 (11.4)	51.7 (7.6)
Philippines (Manila)	825	22.9	53.2 (9.4)	50.9 (10.4)	53.9 (8.9)	46.4 (7.6)	43.2 (8.4)	47.3 (7.1)
Philippines (Nampicuan-Talugtug)	722	28.8	50.1 (7.2)	47.8 (7.4)	51.1 (6.9)	45.6 (7.6)	40.3 (8.9)	47.7 (5.8)
Poland (Krakow)	480	24.1	47.7 (10.2)	44.0 (10.6)	48.9 (9.8)	43.9 (10.6)	36.2 (9.6)	46.4 (9.7)
Portugal (Lisbon)	564	12.6	50.3 (11.4)	45.0 (10.3)	51.1 (11.3)	49.7 (8.6)	43.2 (9.3)	50.6 (8.1)
Saudi Arabia (Riyadh)	609	22.5	50.2 (7.8)	46.7 (8.1)	51.2 (7.4)	48.5 (8.6)	40.7 (8.1)	50.8 (7.3)
South Africa (Uitsig-Ravensmead)	766	28.7	49.0 (9.8)	44.9 (9.7)	50.7 (9.3)	46.5 (9.4)	39.2 (9.6)	49.5 (7.6)
Sudan (Gezeira)	386	7.9	53.3 (10)	47.7 (8.9)	53.7 (10.0)	47.4 (7.5)	42.4 (10.2)	47.8 (7.1)
Sudan (Khartoum)	479	6.8	47.6 (9.2)	46.1 (10.4)	47.7 (9.1)	48.8 (7.4)	42.8 (10.7)	49.2 (6.9)
Sweden (Uppsala)	489	5.5	43.5 (6.8)	45.0 (6.0)	43.5 (6.8)	48.8 (6.6)	40.9 (8.1)	49.2 (6.3)
Trinidad and Tobago	1051	8.5	54.9 (8.4)	50.1 (11.3)	55.4 (7.9)	48.2 (7.1)	39.8 (12.2)	49.0 (5.9)
Tunisia (Sousse)	433	15.4	50.5 (9.7)	47.2 (10.4)	51.1 (9.5)	47.4 (8.6)	41.4 (8.3)	48.5 (8.2)
USA (Lexington, KY)	416	19.6	50.4 (11.1)	42.8 (14.0)	52.3 (9.3)	45.8 (11.4)	35.1 (11.2)	48.4 (9.9)

## Discussion

To the best of our knowledge, this is the first study investigating the association of breathlessness and quality of life across sites in different world regions. This study included a large sample size and sites from five continents as well as high-, middle-, and low-income countries. The design was population-based thus not investigating breathlessness in connection to a specific condition or disease, but with a focus on the general adult population.

This study shows that individuals with breathlessness experience lower quality of life in both t mental and physical components. However, the association between breathlessness and quality of life was stronger for the physical domain. These findings are in line with those reported in samples of the Health Omnibus Survey (HOS) conducted in South Australia^8,9^ and the European Community Respiratory Health Survey (ECRHS) including data from 22 European countries.^12^ Research on measuring quality of life in various populations has shown that a change in 3 to 5 points on the mental and physical subscales of the SF-12 questionnaire can be considered clinically relevant.^21–23^ The magnitude of the association with the physical component score in our study clearly exceed this range. Our data therefore show that breathlessness and physical quality of life are inversely associated. This argument is supported by recent publications showing that being breathless leads to physical deconditioning, muscle loss, limitations in mobility and activities of daily living and reduced sexual activity.^8,24–26^ Mental component scores were also lower in participants with breathlessness in our study, although the association was weaker than for the physical component. This might be explained by the composition of the SF-12 questionnaire. It can be argued that potential limitations in everyday activities caused by breathlessness may have a stronger impact on the physical SF-12 component score based on the questions asked in the questionnaire (e.g., moderate activities such as moving a table, pushing a vacuum cleaner, bowling, playing golf or climbing several flights of stairs – Does your health now limit you in these activities? If so, how much?). As no data on physical activity levels of participants were available, we could not include these in our regression model as a potential confounding factor. In addition, the mMRC scale measures breathlessness related to physical activity, without acknowledging the emotional or social impact of breathlessness and thus potentially leading to a weaker association of breathlessness and the mental quality of life component score in our analyses. Using a more comprehensive tool to quantify dyspnoea such as the Dyspnoea-12 questionnaire^27^ or the Multidimensional Dyspnoea Profile^28^ might lead to a stronger association between breathlessness and the mental quality of life component. Despite some limitations of the tools used to quantify breathlessness and quality of life in this study, it is reasonable to conclude that both physical and mental quality of life domains are negatively associated with the presence of breathlessness.

This study offers data from different world regions and different levels of gross national income. The association between physical component score and dyspnoea was stronger in high-income countries. However, the differences were only to a small extent and there was substantial heterogeneity of results across all sites. This heterogeneity is partly explained by sex and smoking status. Sex differences in the pathophysiology and perception of breathlessness have been described in previous research. Data from the Swedish CardioPulmonarybioImage Study (SCAPIS) and the third European Community Respiratory Health Survey (ECRHS III) showed that lower absolute lung volumes can explain why breathlessness during physical activity is more common in women.^29,30^ In addition, altered respiratory mechanics caused by relatively smaller airway diameters (dysanapsis) and differences in reporting respiratory symptoms may explain why women are more strongly affected by breathlessness than men.^31^ Research on gender differences in COPD and asthma patients has also shown differences in self-reported quality of life.^28,32,33^ Women seem to experience lower mental quality of life compared to men with similar disease severity. Previous research has also shown that smoking is independently associated with breathlessness, even in absence of smoking-related disease.^34^ This might be due to higher airway resistance and reduced oxygen uptake during physical activity leading to an increase in exertional breathlessness in smokers. Furthermore, previous research has shown that smoking is independently associated with reduced quality of life.^35,36^ Smoking can lead to lower physical quality of life based on the mechanisms described above while in addition a lower mental wellbeing increases the likelihood of starting with tobacco smoking and reduces the rate of quitting it.

Although the design of our study was population-based and therefore mainly focused on healthy individuals, the findings may have several implications for clinical practice in people with chronic breathlessness. In a large systematic review of qualitative studies, Hutchinson and colleagues investigated mental coping mechanisms of patients with breathlessness.^37^ They showed that people with chronic dyspnoea may either use engaging mechanisms to cope with their situation, leading to better outcomes and treatment, or disengaging coping mechanisms characterised by self-blame, shame, and social isolation due to their condition. Currow and colleagues found that quality of life is lowest in individuals living with chronic breathlessness for two to six years after symptom onset, while the negative effects of breathlessness on quality of life slightly wear off after this time.^8^ This may come as a surprise, but the authors argue that patients and their environment seem to adapt to the situation over time and sometimes seem to rethink their individual definition of quality of life. As breathlessness and physical wellbeing seem to be strongly associated, clinicians will have to focus on promoting physical activity and rehabilitation services in patients with chronic breathlessness, thus improving their physical functioning. They should further focus on enabling patients in developing coping strategies to reduce the potential negative impact on the patient’s mental quality of life. A possible model for including these different components of physical and mental wellbeing in patient care programs for breathless individuals is the breathing-thinking-functioning (BTF) model.^38^ This clinical model comprises respiratory interventions (e.g., handheld fan, breathing techniques, respiratory muscle training) alongside psychological support (e.g., relaxation techniques, cognitive behavioural therapy) and active rehabilitation and can be used to develop and guide integrated breathlessness management services.

The strengths of this study include, as mentioned earlier, new data on breathlessness from areas of the world that have previously been underrepresented, its population-based design, and the standardised protocol across all sites. However, there are also limitations to this study. The cross-sectional design does not allow conclusions about causality between breathlessness and quality of life, although it is unlikely a worse quality of life would cause breathlessness. Another limitation is that participants were 40 years or older, thus not fully representing the adult general population. Also, even if widely used in epidemiological research, the mMRC breathlessness scale might not properly measure clinically relevant dyspnoea in population-based studies as we argued in the past.^3,4^ Especially not in a global context, as linguistic and societal differences might lead to over- and underestimation of prevalence estimates. The translation of the study questionnaire followed a standardised forward-backward translation process to reduce language bias.^14^ However, linguistic or cultural differences might still limit the comparability of self-reported outcomes across different countries. In addition, no participants from hospitals or nursing homes were included, and it may be argued that a relevant proportion of individuals living with quality-of-life impairment due to chronic breathlessness might be found in these institutions.

## Conclusion

This study offers new data on the association of breathlessness and quality of life including a large number of sites from different world regions. It shows that quality of life is negatively related to the presence of breathlessness, with the association being greater for the physical quality of life component. Further research should focus on investigating the variation in association between quality of life and breathlessness, as we were only able to partially explain the heterogeneity in this study. Future studies might also use longitudinal approaches to follow-up individuals with chronic breathlessness over time to see how changes in breathlessness severity have an impact on quality of life.

## Supplementary Material

Supplement_BOLD_Dyspnoea_QoL_Revision1.docx
