# Supplementary material for: Quality of life associated with breathlessness in the multinational Burden of Obstructive Lung Disease (BOLD) study: A cross-sectional analysis
Source: Pulmonology. 2025 Apr 2;31(1):2470566. doi: 10.1080/25310429.2025.2470566 (PMC11974890; doi:10.1080/25310429.2025.2470566)
Supplement: Supplement_BOLD_Dyspnoea_QoL_Revision1.docx [file TPUL_A_2470566_SM2789.docx]

**Title:** Quality of life associated with breathlessness in the multinational Burden of Obstructive Lung Disease (BOLD) study: A cross-sectional analysis

**Authors:** Alexander Müller, Emiel FM Wouters, Peter Burney_,_ James Potts, Joao Cardoso, Mohammed Al Ghobain, Michael Studnicka, Daniel Obaseki, Asma Elsony, Kevin Mortimer, David Mannino, Rain Jõgi, Rana Ahmed, Asaad Nafees, Maria Fatima Rodrigues, Cristina Bárbara, Rune Nielsen, Thorarinn Gíslason, Hamid Hacene Cherkaski, Karima El Rhazi, Christer Janson, Mahesh Padukudru Anand, Sanjay Juvekar, Herminia Brites Dias, Frits ME Franssen, Dhiraj Agarwal, Sylvia Hartl, Terence Seemungal, Stefanni Nonna Paraguas, Imed Harrabi, Meriam Denguezli, Abdul Rashid, Gregory Erhabor, Mohammed El Biaze, Parvaiz Koul, Daisy JA Janssen, André FS Amaral on behalf of the BOLD Collaborative Research Group.

**Supplementary material**


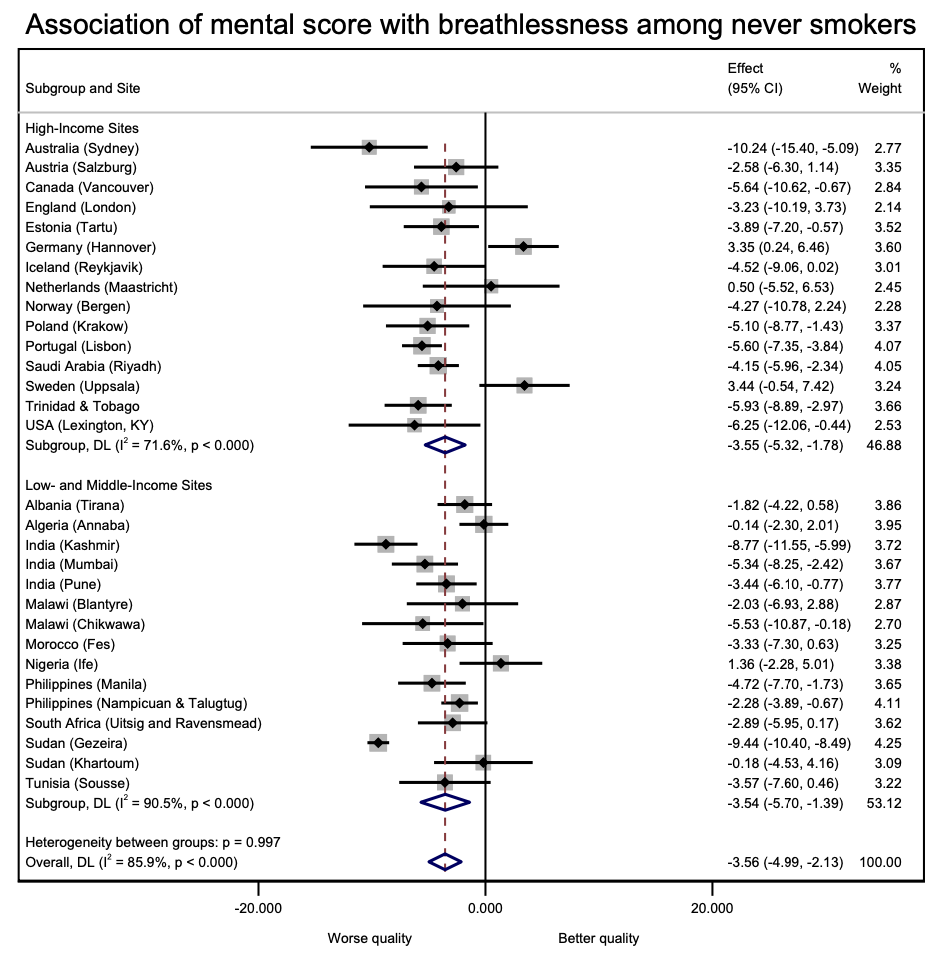

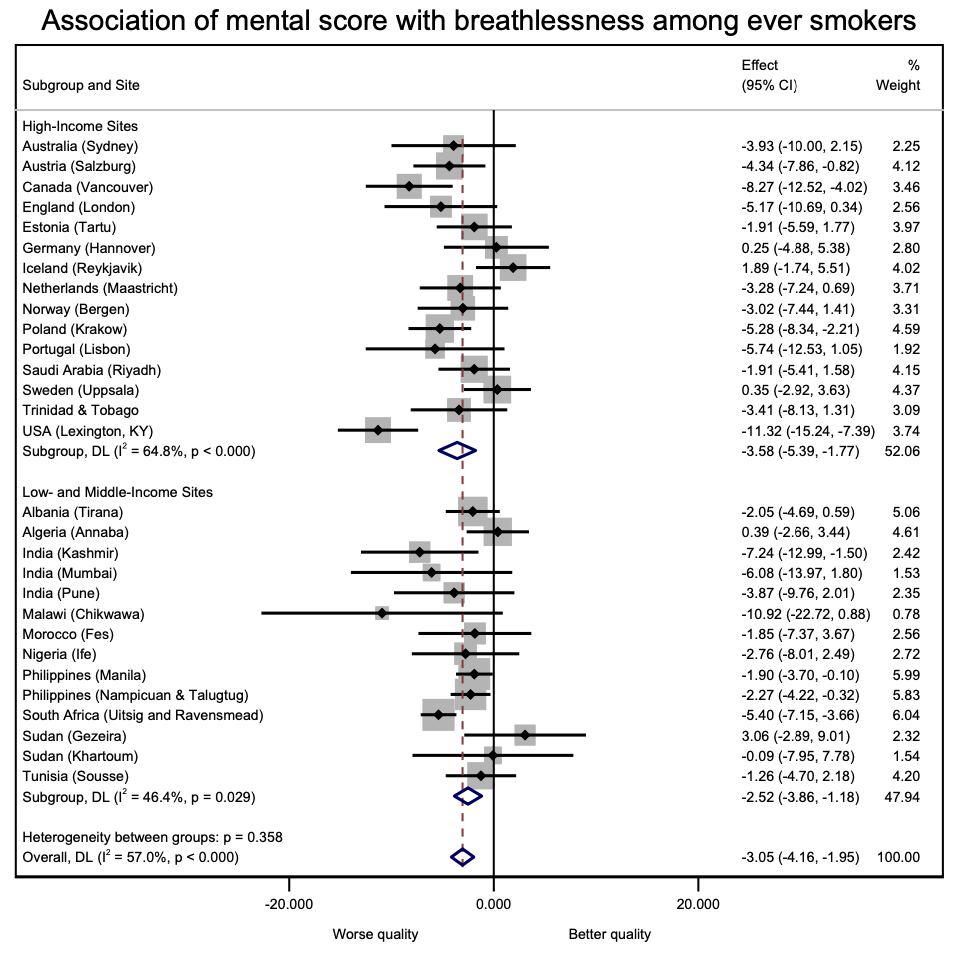


Figure S1: Association of breathlessness and mental SF-12 component score across BOLD sites, stratified by smoking status


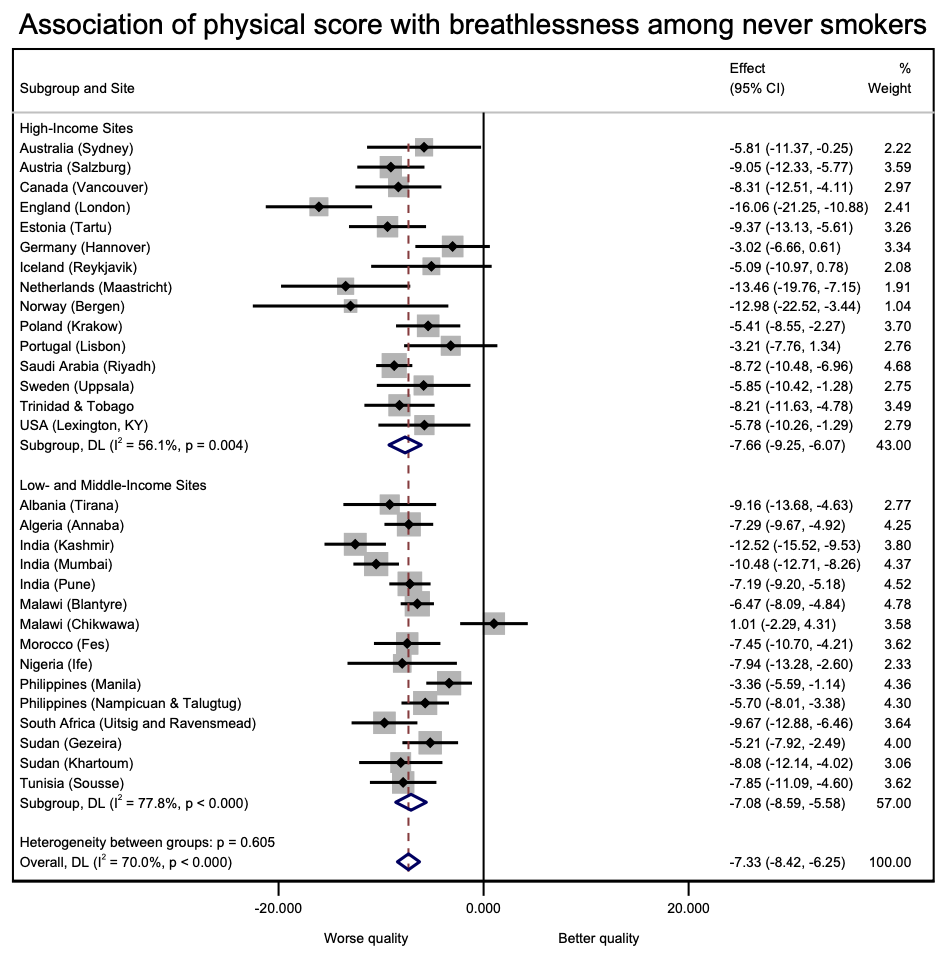

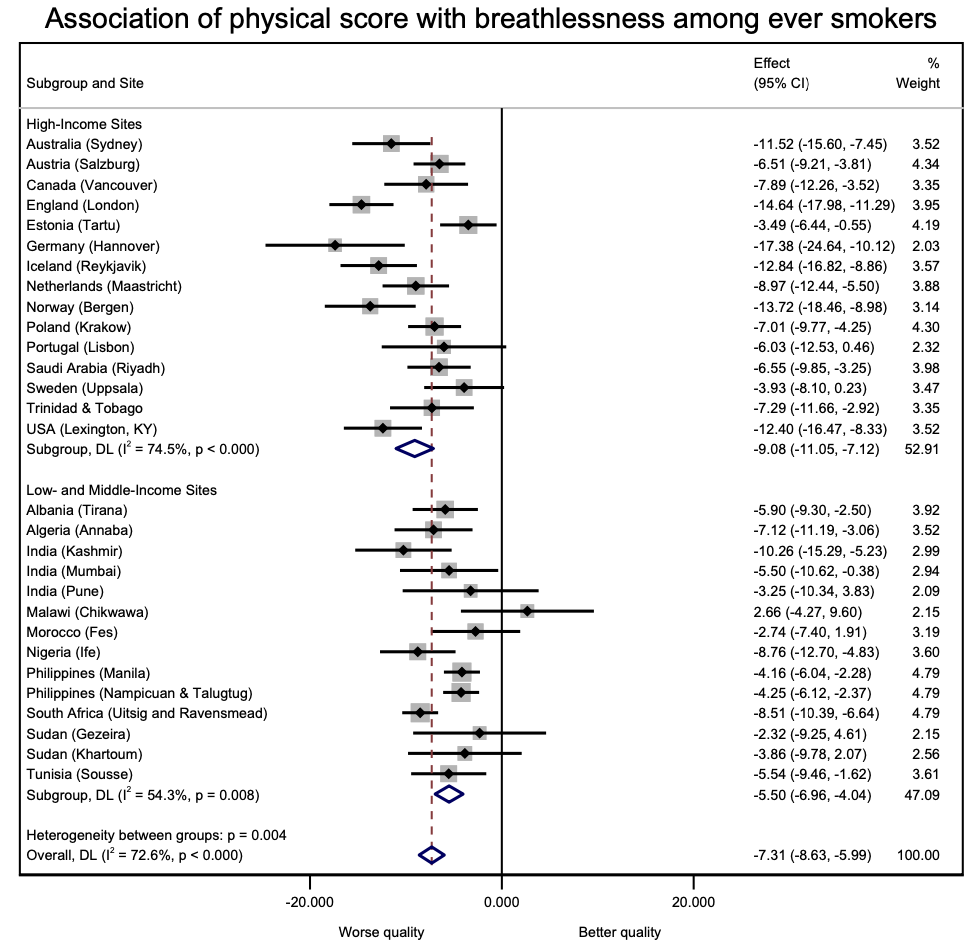


Figure S2: Association of breathlessness and physical SF-12 component score across BOLD sites, stratified by smoking status


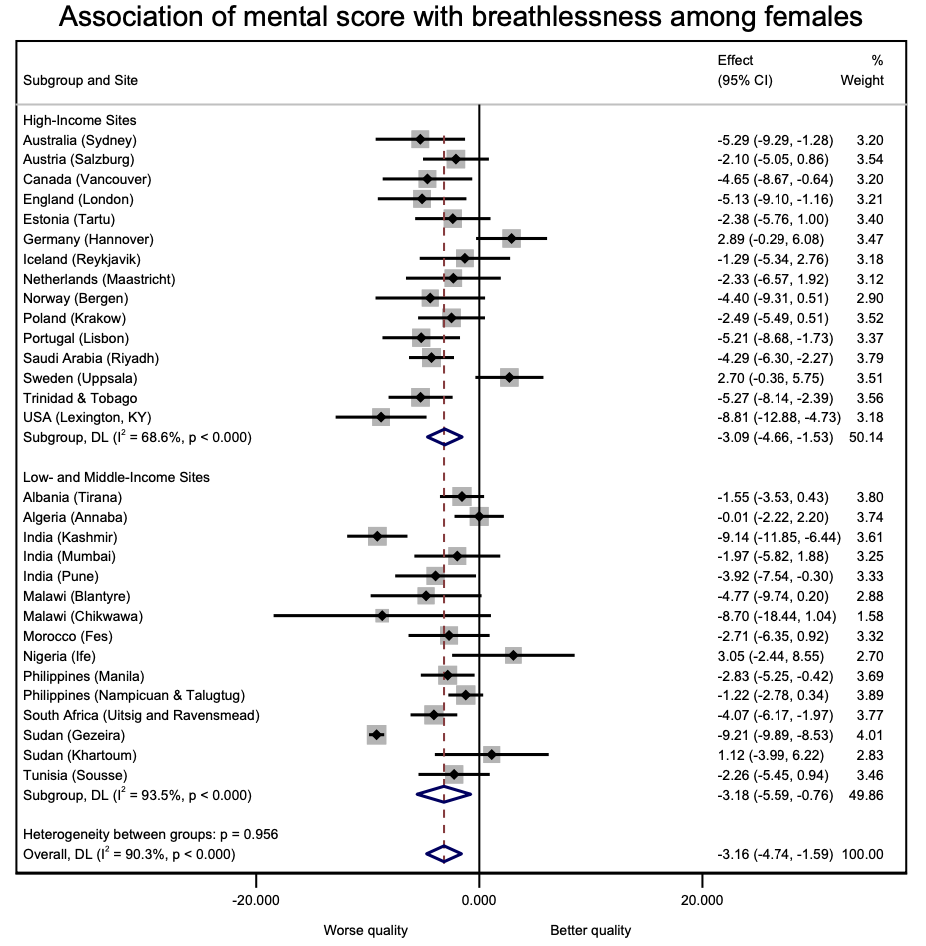

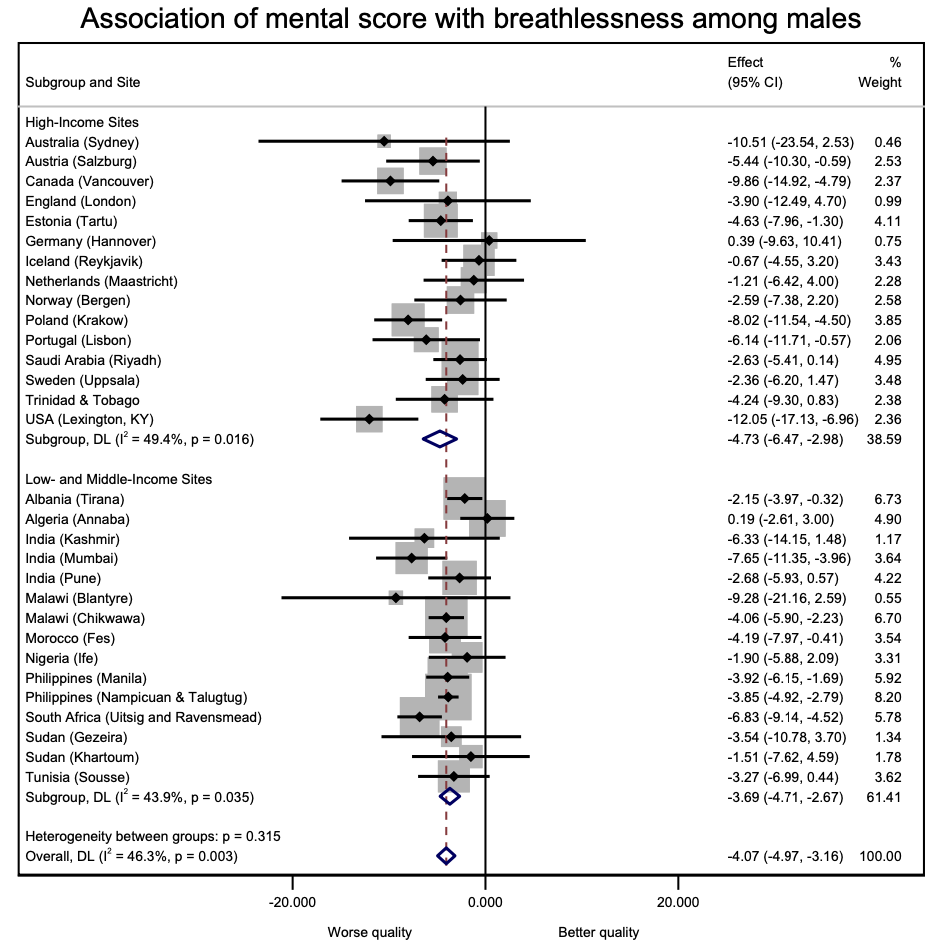


Figure S3: Association of breathlessness and mental SF-12 component score across BOLD sites, stratified by sex


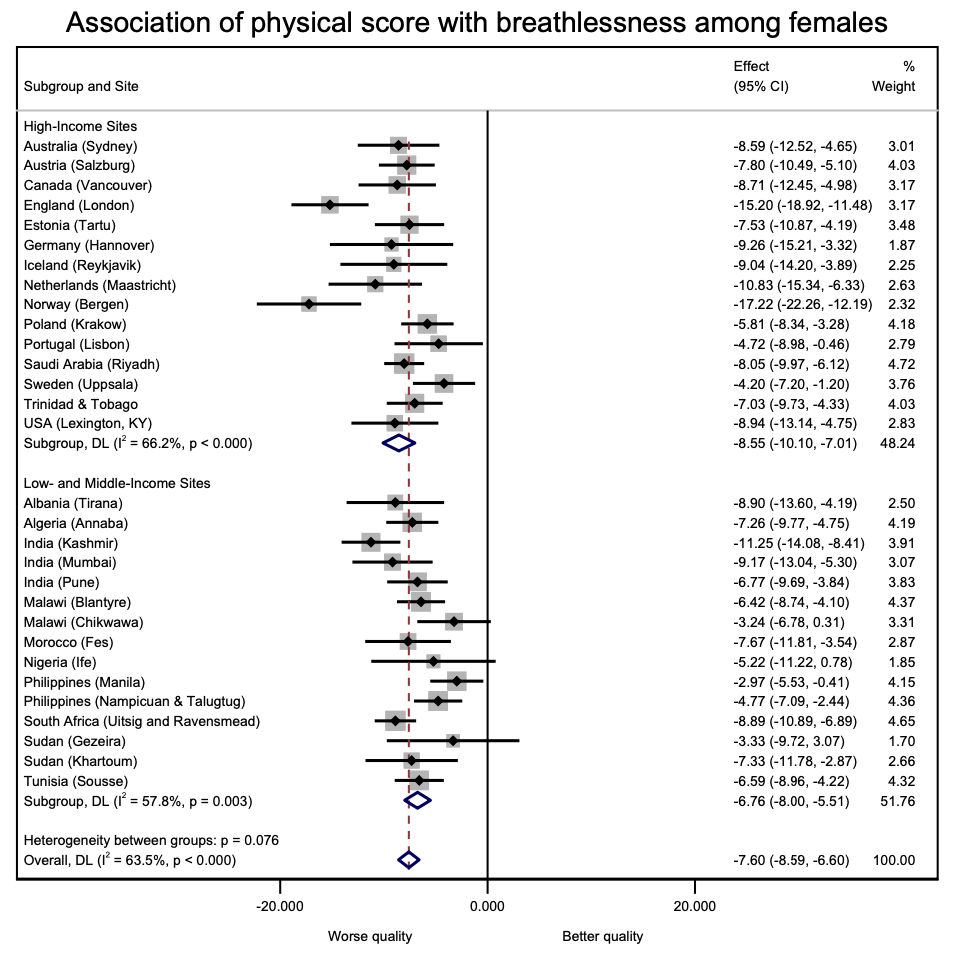

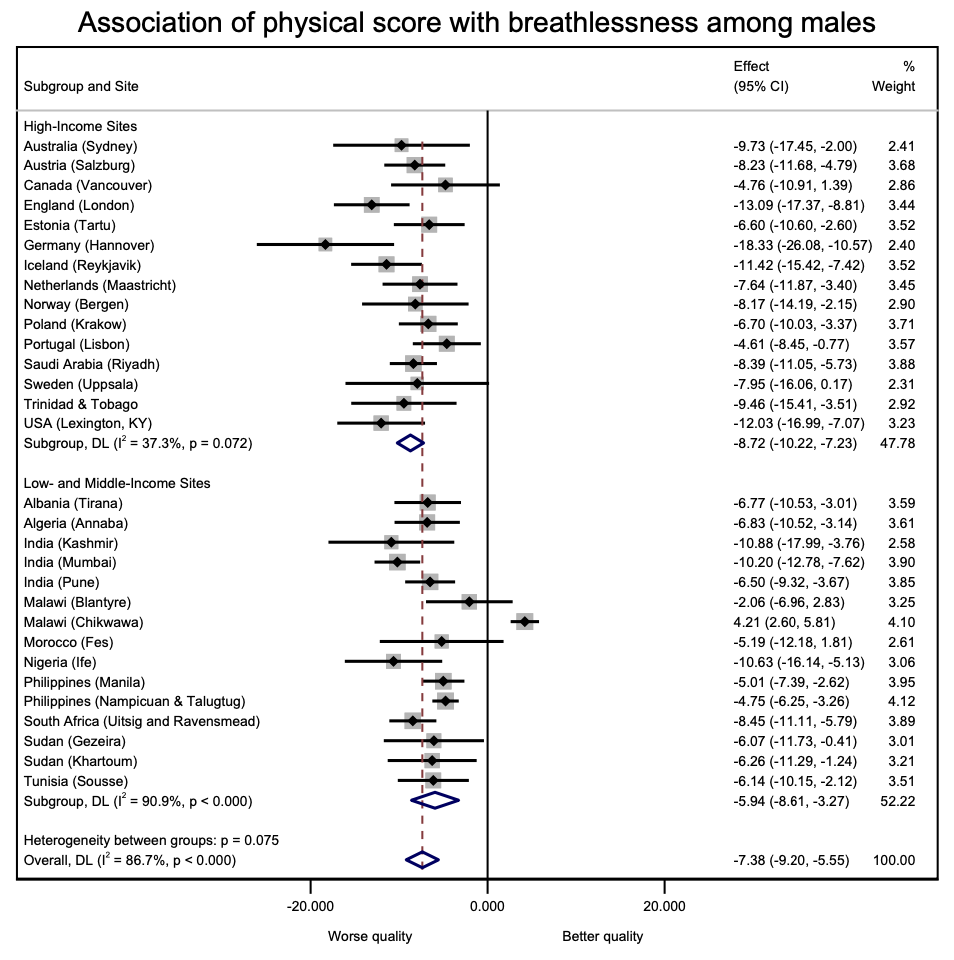


Figure S4: Association of breathlessness and physical SF-12 component score across BOLD sites, stratified by sex
